# Correlations Between Ultrasound Features and Histological Findings in Adenomyosis: A Single-Center Retrospective Study

**DOI:** 10.3390/diagnostics16111586

**Published:** 2026-05-22

**Authors:** Melinda-Ildiko Mitranovici, Dan Costachescu, Septimiu Voidazan, Liviu Moraru, Laura Caravia, Florin Bobirca, Mihai Munteanu, Romeo Micu

**Affiliations:** 1 Department of Obstetrics and Gynecology, Emergency County Hospital Hunedoara, 14 Victoriei Street, 331057 Hunedoara, Romania; mitranovicimelinda@yahoo.ro; 2 Department of Anatomy, University of Medicine, Pharmacy, Sciences and Technology, 540142 Targu Mures, Romania; septimiu.voidazan@umfst.ro (S.V.); liviu.moraru@umfst.ro (L.M.); 3 Department of Radiology and Medical Imaging, University of Medicine and Pharmacy Victor Babes, 2 Eftimie Murgu Square, 300041 Timisoara, Romania; 4 Faculty of Medicine, “Carol Davila” University of Medicine and Pharmacy, 050474 Bucharest, Romania; laura.caravia@umfcd.ro (L.C.); bobirca@umfcd.ro (F.B.); 5 Faculty of Electrical Engineering, Technical University, George Baritiu Street, 400394 Cluj Napoca, Romania; mihai.munteanu@ethm.utcluj.ro; 6 Department of Obstetrics and Gynecology, University of Medicine and Pharmacy Iuliu Hatieganu, 400347 Cluj-Napoca, Romania; romeomicu@hotmail.com

**Keywords:** adenomyosis, transvaginal ultrasound, Doppler, histology, three-dimensional ultrasound

## Abstract

Adenomyosis is a benign gynecologic condition characterized by ectopic endometrial glands and stroma present within the myometrium. **Background/Objectives:** The gold standard in diagnosis is the histology of hysterectomy specimens. Due to the heterogeneity of this disease, there is a lack of valid classification. The most important symptoms are chronic pelvic pain and abnormal uterine bleeding, followed by infertility. Noninvasive diagnostic tools have been sought, with ultrasound being a valuable option. The objective of our study was to evaluate the correlation of transvaginal ultrasound, used in addition to three-dimensional ultrasonography and Doppler, with the histology of adenomyosis. **Methods:** An observational retrospective study was conducted between January 2015 and November 2018 on 160 women with adenomyosis managed by hysterectomy. All patients underwent transvaginal sonography combined with 3D and Doppler sonography. **Results:** Comparing the location of adenomyosis in the myometrium observed using ultrasound with histological findings, a statistically significant correlation was observed (*p* = 0.0001). Symptoms were associated with the location of the lesions, heavy period in internal adenomyosis (*p* ≤ 0.001), and infertility (*p* = 0.001), while pelvic pain was observed in external adenomyosis (*p* = 0.03). Deep endometriosis was associated with external adenomyosis (*p* = 0.001). An ill-defined junctional zone was observed via Doppler investigation in internal adenomyosis (*p* = 0.0001), also correlated with the symptoms. Histology confirmed all cases of adenomyosis, with statistically significant similarities regarding pattern, location, and depth (*p* < 0.001). **Conclusions:** The increasing use of 3D and Doppler evaluations enhances TVUS importance, and 3D TVUS offers high diagnostic capacity for adenomyosis, in accordance with histological findings. This procedure facilitates the adoption of therapeutic modalities other than surgery with uterus preservation.

## 1. Introduction

Adenomyosis is a benign gynecologic condition where endometrial-like tissue is distributed within the uterine myometrium. The pathogenesis is characterized by fibrosis, inflammation, and angiogenesis, which are not fully understood. This disease is observed in women between 35 years of age and menopause [1,2,3], and its prevalence varies from 5% to 70% due to the heterogeneity of this disease and the lack of a valid classification [4]. The typical symptoms, characterized by heterogeneity and poor specificity, are abnormal uterine bleeding (AUB) or pelvic pain, dysmenorrhea, and dyspareunia or infertility; however, some women are asymptomatic [1,5,6,7]. Adenomyosis often coexists with endometriosis or fibroids [6,7,8,9,10], which could explain the link between dysmenorrhea and disease severity. Hemosiderin deposition into the adenomyotic lesions was observed in the case of deep penetration caused by bleeding of the ectopic endometrium, which may reflect the severity of adenomyosis [2,11].

The gold standard in diagnosis is histopathology, usually after hysterectomy, but currently, adenomyosis may also be diagnosed by imaging techniques. However, the diagnostic criteria are still not universally agreed upon, and the diagnostic process for adenomyosis is challenging [6,7]. Histology-based classifications focus on disease location or extent [8], while Hulka et al. described “adenomyoma”, which is a new category of focus [12]. Currently, although adenomyosis may also be diagnosed via imaging techniques, the diagnostic criteria are still not universally agreed upon, and the diagnostic process for adenomyosis is challenging [6,7]. Classification systems based on ultrasound and MRI have been proposed but have not gained clinical validation [13]. The definition of adenomyosis is confusing and heterogeneous. This is why a collaboration between ESGE, ESHRE, and WES was mandatory to provide a coherent terminology, diagnosis, and classification of adenomyosis [13]. Regarding the ultrasound classification of adenomyosis, the Morphological Uterus Sonographic Assessment (MUSA) group criteria, which were revised in 2021, aimed at providing uniform guidance for identifying adenomyotic lesions [14,15,16]. A standardized reporting system is thus needed. Color Doppler, two- (2D) and three-dimensional (3D) transvaginal ultrasound imaging (TVS), and magnetic resonance imaging (MRI) should be offered before hysterectomy [4,5,17,18,19,20]. It has also been demonstrated that these imaging findings do not reflect symptom severity [21,22]. Ultrasound and MRI-based diagnosis should be correlated with histopathological features [6,8].

Clinical features are also reported to be associated with different depths of adenomyosis, such as severe dyspareunia, which was observed in the presence of a poorly defined junctional zone (JZ). In addition, patients with abnormal uterine bleeding (AUB) were discovered in women with interrupted JZ. Diffuse adenomyosis was associated with older age, a greater prevalence of infertility, and AUB [15].

Only one recent TVUS-based classification has been validated and correlated with the clinical findings in the MUSA system, but it is not widely accepted [8,18]. It is also correlated with histology, and the outcome after treatment depends on the disease extent [8]. It is mandatory to differentiate adenomyosis from myomas, as they mimic each other in clinical presentation [10,23].

This study compares ultrasound diagnostic criteria of adenomyosis, clinical signs and symptoms, and histopathological features of adenomyotic lesions. The efficacy of combining TVUS, 3D, and Doppler ultrasound was also assessed. The location and pattern of the lesions were considered and the clinical relevance was assessed.

## 2. Materials and Methods

This is a retrospective observational study including women with adenomyosis managed via hysterectomy and was conducted from January 2015 to November 2018 in the Department of Obstetrics and Gynecology, Emergency County Hospital, Hunedoara. The protocols for this manuscript were approved by the Ethics Committee of Al Simionescu County, No. 3510/18 February 2025, for the analysis of the retrospective data. After reviewing the protocol, the Ethics Committee approved the waiver of informed consent for the retrospective chart and pathology review conducted in 2025 regarding the 2015–2018 data. Data extraction and chart review were performed between 20 February 2025 and 30 March 2025. In this manuscript, we include the ultrasound data analysis and the reevaluation of histological pieces according to the new criteria.

From a total of 206 patients diagnosed with adenomyosis, 160 patients who underwent hysterectomy were included. The inclusion criteria used in our study were increased uterine volume, prolonged and abnormal uterine bleeding, and chronic pelvic pain. Patients who did not undergo hysterectomy and those who received medical treatment, refused surgery, or did not sign the informed consent form for research were excluded. Written informed consent for both intervention and research was obtained from all patients included in our research. For the intervention, it was signed upon admission to the hospital, and for the study, it was sent by email. Those who did not sign the informed consent form for research were excluded. Those for whom data were not correctly collected were also excluded from our study.

Detailed clinic demographic data were collected. Symptoms such as pelvic pain, abnormal uterine bleeding, and infertility were recorded. A visual analog scale, where the extremes were no pain (score of 0) and pain as bad as it could be (score of 10), was used to grade pelvic pain severity. We included patients with a pain score > 6 on this scale. The Pictorial Blood Loss Assessment Chart was used to assess the volume of blood loss during each menstrual period. The number of pads was quantified, and points were assigned for bleeding and were categorized as follows: zero points (no pad was used), one point (1–3 pads), two points (4–6 pads), three points (7–9 pads), four points (10–12 pads), five points (13–15 pads), six points (16–18 pads), seven points (19–21 pads), eight points (22–24 pads), nine points (25–27 pads), and ten points (≥28 pads). Patients with a score higher than 6 points on this scale were included in our research. Infertility was considered only in patients who had attempted to become pregnant without success and in whom no other diagnoses were found except adenomyosis. It was assessed through TVUS and clinical history, the explanation for infertility being an altered intrauterine environment with chronic inflammation and altered muscle contractions.

All patients were evaluated via detailed 2D transvaginal ultrasound and 3D volume acquisition of the entire uterus, before hysterectomy. Ultrasound examinations were performed and documented by two experts using a Voluson S10 Expert sonography machine (GE Healthcare, Milwaukee, WI, USA) via a transvaginal ultrasound. MUSA criteria were used to diagnose adenomyosis [24], and its sonographic features were noted. We used the multiplanar coronal and longitudinal views, and the following sonographic signs were reported: JZ ill-definition, bulky uterus, and myometrial heterogeneity with cysts. It is not a classification or staging system, but defines a descriptive reporting framework. We then used clinical reporting based on pattern (diffuse and focal) and depth, close to JZ and inner myometrium, described as internal adenomyosis or external adenomyosis, which affects the outer myometrium/subserosa [25]. Doppler velocimetry was also performed to diagnose adenomyosis, and the morphology, tumor vascular pattern, and blood flow impedance of the uterine tumors were measured. While interobserver agreement is heavily influenced by the operator’s ability to acquire the image, not just interpret it, we decided to use only the period of time in which the patients were investigated by the best sonographer who recorded detailed data.

Tissue samples were collected immediately after hysterectomy, and all the tissues were fixed in 10% neutral-buffered formalin for a minimum of 24 h before processing. Microscopic evaluations were performed by two pathologists. The examination of specimens followed the institutional routine criteria and was then re-reviewed according to later consensus definitions in accordance with the Delphi Group Consensus Statement [26]. The samples were reevaluated by two experienced pathologists, and any discrepancies were resolved by reviewing the slides together to increase diagnostic accuracy.

While no single, universally accepted, or standardized classification system exists, recent international consensus and established guidelines provide key criteria for diagnosis and reporting. A 2025 international Delphi consensus, based on 31 experts, provided updated recommendations to reduce variability in pathological diagnosis: diagnostic thresholds—2 mm depth of glands/stroma or 1/3 of myometrial thickness with a standard description of adenomyoma and focal, extensive superficial, and deep adenomyosis [26]. The histopathological assessment focused on the depth of invasion: if close to the endometrial–myometrial junction, it was considered internal (superficial), if close to the peritoneum, it was considered external (deep); the aspect of junctional zone, histological pattern (diffuse, focal, or adenomyoma).

The patients were divided into two groups, based on TVUS: those with external adenomyosis, meaning lesions found in the external part of the myometrium facing the peritoneum (*n* = 69), and those with internal adenomyosis, near the endometrium (*n* = 91). The TVUS findings were blindly correlated with the histopathological results. To ensure accuracy, the study only used data from the most experienced sonographer, and pathological discrepancies were resolved by joint review.

### Statistical Analysis

The normality of the distribution of the numerical variables was assessed using the Kolmogorov–Smirnov test. Numerical data are expressed as the mean (standard deviation) or as the median (interquartile range) for normally and non-normally distributed data, respectively.

The chi-squared test was used to compare categorical variables. An inter-rater agreement statistic (K, Kappa) was calculated with a 95% confidence interval. K is 1 when there is perfect agreement between the classification systems; K is 0 when there is no agreement better than chance; K is negative when agreement is worse than chance. A value of K = (0.61–0.80) means good strength of agreement. A value of K = (81–1.00) means a very good strength of agreement. *p*-values lower than 0.05 were considered statistically significant. ROC performance was analyzed, and statistical analysis was performed using SPSS (IBM SPSS Statistics for Mac 2024, Version 30.0, Armonk, NY, USA: IBM Corp).

## 3. Results

### 3.1. Demographic Characteristics

We compared the ultrasound findings regarding the internal versus external location of adenomyosis with the histological findings. Women with an internal location had similar ages and weights to women with an external location of adenomyosis on the scan. More than 90% of women were married and had a stable relationship.

There was a significant difference between multiparous and nulliparous women, especially in the group with an external location of adenomyosis (Table 1).

### 3.2. Ultrasound Versus Histology

We analyzed the localization of the lesions, their pattern (diffuse or focal), junctional zone, and Doppler aspects of the vascularization within the adenomyosis lesions.

#### 3.2.1. Adenomyosis Location

Comparing similarities between histological and ultrasound aspects, we observed that out of 91 patients with internal localization of adenomyosis detected at ultrasound, 79 (86.8%) had histological confirmation. We applied the chi-squared test, which revealed a statistically significant association (*p* = 0.0001), and weighted Kappa agreement was also very good at 82.5 (CI95% 73.8 to 91.2). In Table 2, we show the results according to the diffuse or focal lesion pattern.

**Figure 1:**
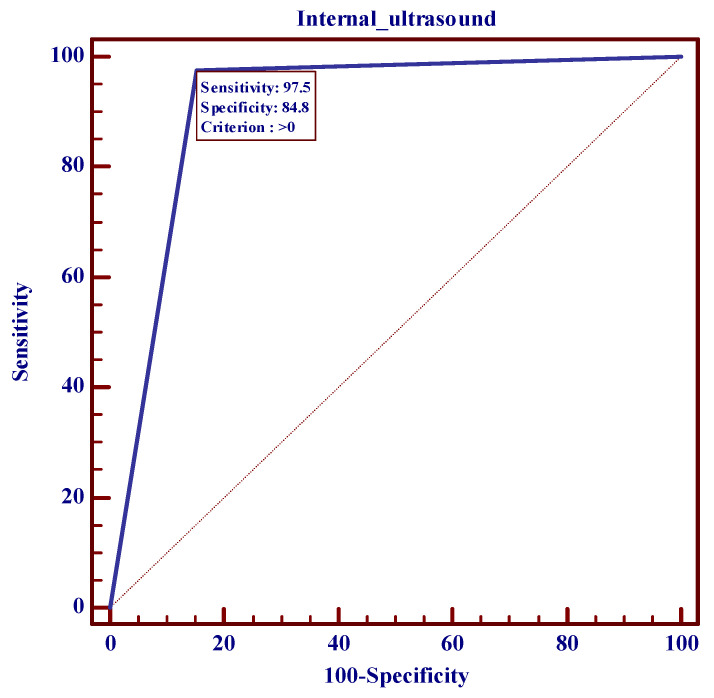
ROC curve analysis for internal adenomyosis according to ultrasound.

Based on ultrasound and histological criteria, we identified a sensitivity of 97.53% (CI95%: 91.4–99.7) and specificity of 84.81% (CI95%: 75.0–91.9) for internal adenomyosis, with an AUC of 91.2% (CI95%: 85.7–95.1) (Figure 1).

The same comparison between histology and ultrasonography was performed for the external localization of the lesions: Out of 69 patients with external lesions detected via ultrasound, 58 (84.1%) also had external lesions on histological analysis. We applied the chi-squared test, which revealed a statistically significant association (*p* = 0.0001), and weighted Kappa agreement was also very good at 78.1 (good) (CI95% 68.4 to 87.9). In Table 3, we show the results according to the diffuse or focal lesion pattern.

**Figure 2:**
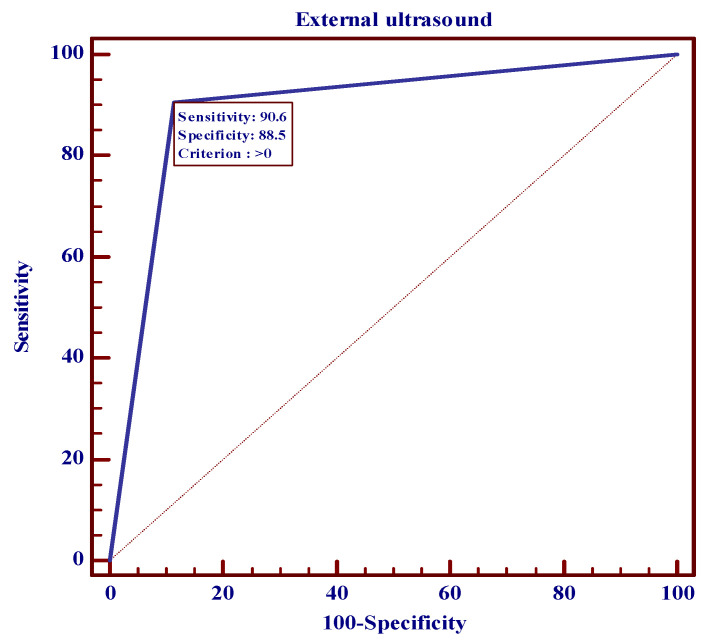
ROC curve analysis for external adenomyosis based on ultrasound.

Based on ultrasound and histological criteria, we identified a sensitivity of 90.62% (CI95%: 80.7–96.5) and specificity of 88.54% (CI95%: 80.4–94.1) for external adenomyosis, with an AUC of 89.6% (CI95%: 83.8–93.9) (Figure 2).

This shows a good match between the location of the lesions found using ultrasound and that using histology.

#### 3.2.2. Altered Junctional Zone

Another significant finding is that the disrupted junction zone was clearly seen on the scan in most of the cases with internal adenomyosis. Regarding junctional zone (JZ) disruption or alteration, we found a statistically significant match between TVUS and histological description. Out of 120 patients with ultrasound confirmation, 106 (88.3%) had histological confirmation. The weighted Kappa agreement index was 72.3 (good) (CI95% 60.5 to 84.1).

**Figure 3:**
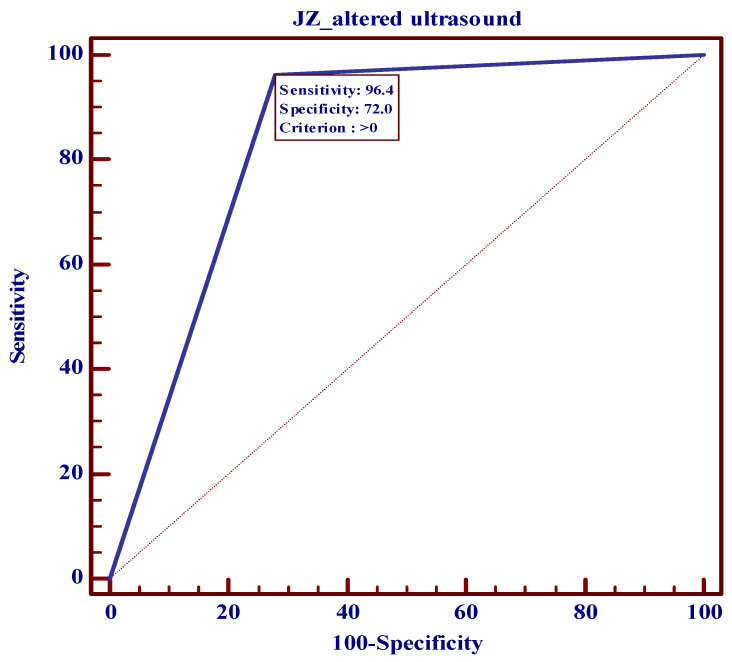
ROC curve analysis for ZJ-altered ultrasound. JZ junctional zone.

Based on ultrasound and histological criteria, we identified a sensitivity of 96.36% (CI95%: 91.0–99.0) and specificity of 72.0% (CI95%: 57.5–83.8) for the altered JZ appearance of adenomyosis, with an AUC of 84.2% (CI95%: 77.6–89.5) (Figure 3).

#### 3.2.3. Doppler Findings

The Doppler analysis did not show any difference between the TVUS and histology, with a *p*-value of 0.45. We found randomly distributed vessels with intra-tumoral Doppler signals in a proportion of >98% in both locations of adenomyosis, which indicates increased vascularity, but with low correlation to histological findings. In addition, a significant percentage of the adenomyotic lesions had a pulsatility index (PI) > 1.17 in the arteries within the lesions.

### 3.3. Symptoms and Associated Diseases

Women with internally located adenomyosis had heavier periods, and there was a statistically significant difference when compared with women with externally located adenomyosis (*p* = 0.001). The rate of female infertility was significantly associated with internal adenomyosis, close to the endometrium (*p* = 0.001). Pelvic pain was observed in the case of external lesion location (*p* = 0.03). Two associated diseases were found: leiomyomas and deep endometriosis. While leiomyomas were found especially in cases of internal endometriosis, although not significant statistically, a more important association with deep endometriosis was found in the case of external adenomyosis location (*p* = 0.001) (Table 4).

We also analyzed the influence of disrupted JZ on the appearance of symptoms. We observed a statistically significant link between altered junctional zone and abnormal uterine bleeding (*p* = 0.0001), pelvic pain (*p* = 0.047), and female infertility (*p* = 0.0001) (Table 5).

Histology confirmed all cases of adenomyosis, with a statistically significant accuracy of location (*p* = 0.0001 in both internal and external adenomyosis found with TVUS) (Table 3). Adenomyoma was present in one out of six cases in both groups.

## 4. Discussion

Approximately 20% of women are affected by adenomyosis, with diverse manifestations. Because of its heterogeneity, adenomyosis remains understudied. Moreover, it is frequently associated with other gynecologic diseases, such as leiomyomas and deep endometriosis. However, the lack of specific treatments for adenomyosis represents a challenge in clinical management [27].

The gold standard in diagnosing adenomyosis is histopathological examination, but imaging techniques have recently gained importance when diagnosing this pathology [4]. Imaging tools potentially provide better accuracy in determining the lesion’s volume, location, and pattern [8,10]. While transabdominal sonography has limited value, transvaginal ultrasound and magnetic resonance have high diagnostic accuracy [4,28,29]. According to Jain, MRI has a higher accuracy than TVS [4]; the most sensitive sign is a JZ thickness ≥ 12 mm, and the most specific MRI feature is the hyperintense myometrial focus [4,30]. TVUS is a widely available and low-cost diagnostic technique, but a universally useful classification is urgently needed for ultrasounds [8,10]. In Bazot’s study on 129 women with adenomyosis scheduled for hysterectomy, TVS has shown a high degree of accuracy compared to transabdominal ultrasound (TAS). The sensitivity and specificity in TAS were 80.9% and 38.4% compared to 100% and 83.3% in TVS, respectively [28].

In El Kattan’s study of 48 women diagnosed with adenomyosis through TVS (out of 52 included), only 37 patients were histologically confirmed. Myometrial cysts and ill-defined JZ were sonographic signs significantly associated with adenomyosis [18]. In Exacoustos’s study, myometrial cysts were the most specific 2D-TVS feature, and heterogeneous myometrium was the most sensitive one, while for 3D-TVS, the most specific sign was JZ alteration [5]. In our study, a statistically significant location accuracy was found (*p* = 0.0001) [Table 2 and Table 3]. In addition, a significant correlation between TVUS and histological findings has been found regarding the altered junctional zone (*p* = 0.0001). Compared to histology, ultrasound showed an AUC of 91.2% (CI95%: 85.7–95.1) for internal adenomyosis and 89.6% (CI95%: 83.8–93.9) for external adenomyosis.

Diagnostic sensitivity was increased by introducing three-dimensional ultrasound [23]. Our study assessed the altered JZ appearance via 3D ultrasound to identify adenomyosis; when compared against histological criteria, 3D ultrasound demonstrated a sensitivity of 96.36% (CI95%: 91.0–99.0) and specificity of 72.0% (CI95%: 57.5–83.8), with an AUC of 84.2% (CI95%: 77.6–89.5).

There was no difference between the focal and diffuse patterns of these lesions between the two groups. A disrupted junction zone was a major finding in the TVUS of adenomyosis, more clearly seen in internal adenomyosis and more accurately described with 3D-TVUS. Moreover, an ill-defined junction zone was present in internally located adenomyosis with statistical significance (*p* = 0.0001).

Our study showed a high correlation between TVUS/3D and histological features of adenomyosis regarding localization/JZ/pattern, with important relevance in clinical use, for example, the use of embolization or ultrasound for adenomyosis-targeted treatment.

In El Kattan’s study of 48 women diagnosed with adenomyosis, the uterine artery pulsatility index was measured but exhibited no significant changes in the case of adenomyosis [18]. In our study, the Doppler ultrasound did not show any difference between the TVUS and histology, with a *p*-value of 0.45, while randomly distributed vessels in a proportion of >98% in both locations of adenomyosis were observed, with a high pulsatility index > 1.17. Three-dimensional color Doppler ultrasound showed efficacy in adenomyosis diagnosis; enhanced echogenic spots were observed within the myometrium, with short or short-branch blood flow signals in the lesions [24,29].

The introduction of the Morphological Uterus Sonographic Assessment (MUSA) criteria significantly improved adenomyosis diagnosis; however, this tool remains challenging [7]. This observation is based on comparing a noninvasive imaging technique with postoperative histology examination of uterine specimens [23]. A subjective interpretation can lead to misdiagnosis of adenomyosis. Despite this observation, the accuracy of TVUS is reported to be high [30], as our study showed.

However, treatment is based on symptom severity rather than the location or pattern of the lesions [21]. According to our study, the location of adenomyosis significantly influenced symptoms, and heavier bleeding was found in internally located adenomyosis (*p* = 0.0001); female infertility was also observed in internally located adenomyosis in a statistically significant proportion (*p* = 0.0001). On the other hand, pelvic pain was a common symptom in external adenomyosis (*p* = 0.03). An important finding was the link between an altered JZ diagnosed through 3D TVUS and symptoms: an altered junctional zone was highly associated with abnormal uterine bleeding (*p* = 0.0001), pelvic pain (*p* = 0.047), and infertility (*p* = 0.0001). Our study confirms what has been emphasized in the literature, namely, that some of the symptoms are positively correlated with the disease topography. Dysmenorrhea, female infertility, and abnormal uterine bleeding were observed in women with diffuse and internal adenomyosis, but also in those with focal adenomyotic lesions. Two ultrasound markers are mandatory to diagnose adenomyosis [21,23,24]. In Jha’s study, heavy menstrual bleeding was associated with interrupted JZ and heterogeneous myometrium, and pelvic pain was significantly associated with myometrial cysts [24]. In his research on adenomyosis among adolescents evaluated through ultrasound, Exacoustos also showed its significant correlation with clinical symptoms [5].

Many studies have shown that internal adenomyosis is more frequent than external and affects older women who have not experienced infertility [21,31,32,33]. In our study, internal adenomyosis was more frequent but did not reach statistical significance, with a disrupted and ill-defined junction zone, characteristic especially of internal adenomyosis (*p* = 0.0001). Internal adenomyosis affects the myometrium diffusely and coexists with leiomyoma more frequently [21,31,32,33].

With reference to clinical features, on the contrary, other studies showed no significant differences between internal and external adenomyosis groups [21,31,32,33]; in contrast, in our research, we observed heavier bleeding in women with internally located adenomyosis, which was statistically significant when compared to women with externally located adenomyosis (*p* = 0.0001). Other authors reported severe dyspareunia associated with a poorly defined junctional zone (JZ) (*p* = 0.023) or abnormal uterine bleeding correlated with interrupted JZ; infertility was also found in diffuse adenomyosis [15]. In our study, we observed the same correlation with ill-defined JZ.

A high association between adenomyosis and endometriosis was also documented. Dysmenorrhea was the most common sign, and it was observed in women in whom the outer myometrium was affected. Abnormal uterine bleeding was observed in the case of diffuse adenomyosis or in patients with external adenomyotic lesions [5]. We also observed an important association between endometriosis and externally located adenomyosis (*p* = 0.001). Even if we encountered leiomyomas in patients with adenomyosis, their presence was not influenced by the location of adenomyotic lesions. The relationship between adenomyosis and endometriosis was evaluated in Alborzi’s study. In a study of 154 women with adenomyosis coexisting with endometriosis, diffuse adenomyosis was more frequently associated with deep endometriosis [34]. According to Kobayashi, the coexistence of endometriosis represented an independent predictor of external adenomyosis [21]. However, these two diseases share common features [33], and the presence of deep endometriosis and infertility is frequent in external lesions according to the literature [16,21,31,32,33].

However, adenomyosis remains an under-recognized condition, and definitive diagnosis is possible only through histology after hysterectomy [34]. According to several studies and our research, which aligns with the literature, transvaginal ultrasound associated with Doppler investigation is a valuable option for those wishing to preserve fertility.

The limitations of our study include the fact that it was an observational retrospective study, with no possibility of intervention. The inclusion only of patients who underwent hysterectomy may have introduced selection bias, despite efforts to ensure comprehensive data collection. It was also a single-center study, which is accompanied by bias and decreased reproducibility. The strength of our study lies in the large number of patients included (160) and the techniques that facilitated high-quality evaluation. Regarding novelty, our study included 3D and Doppler techniques, which increased the quality of adenomyosis evaluation. Moreover, two pathologists checked the histological samples, and histology confirmed all cases of adenomyosis. The statistically significant location accuracy (*p* = 0.0001) in both internal and external adenomyosis found with TVUS has important clinical relevance, helping health providers to preserve the uterus and specifically target lesions through different methods.

## 5. Conclusions

The association between lesion location, as diagnosed through TVUS, and symptoms opens up a new avenue in adenomyosis management. The increasing adoption of 3D evaluations enhances the clinical utility of TVUS, and 3D TVUS offers high diagnostic capacity for adenomyosis, in accordance with histological findings, while Doppler examination has not proven its value. In our study, we emphasize the clinical relevance of sonographic signs for diagnosing adenomyosis, facilitating the adoption of therapeutic modalities other than surgery with uterus preservation. Our research requires external validation. Future multicenter prospective studies incorporating standardization should be performed to verify the above results and refine risk prediction models.

## Figures and Tables

**Table 1:** Comparison between ultrasound findings and demographic data.

Variable	Internal Location TVUS (*n* = 91)	External Location TVUS (*n* = 69)	*p*-Value
Age (years)	50 ± 10.4	52.3 ± 8.6	0.19
BMI	31 ± 9.7	32 ± 8.6	0.14
Married, *n* (%)	85 (93.4)	64 (92.8)	0.55
Multiparous, *n* (%)	55 (60.4)	68 (98.6)	**<0.001**

BMI, body mass index; TVUS, transvaginal ultrasound. A *p*-value under 0.05 is considered significant. The bold highlights the statistically significant result.

**Table 2:** Comparison between histological and ultrasound aspects of internal adenomyosis.

Pattern	*p* Value	Histological Location		Location TVUS		Total
				External	Internal	
Diffuse	*p* = 0.0001	Internal (superficial) histology	No	31 (96.9%)	1 (2.0%)	32 (39.0%)
			Yes	1 (3.1%)	49 (98.0%)	50 (61.0%)
		Total		32 (100.0%)	50 (100.0%)	82 (100.0%)
Focal	*p* = 0.0001	Internal (superficial) histology	No	36 (97.3%)	11 (26.8%)	47 (60.3%)
			Yes	1 (2.7%)	30 (73.2%)	31 (39.7%)
		Total		37 (100.0%)	41 (100.0%)	78 (100.0%)
Total	*p* = 0.0001	Internal (superficial) histology	No	67 (97.1%)	12 (13.2%)	79 (49.4%)
			Yes	2 (2.9%)	79 (86.8%)	81 (50.6%)
		Total		69 (100.0%)	91 (100.0%)	160 (100.0%)
Weighted Kappa				0.825		
95% CI				0.738 to 0.912		

Inter-rater agreement (Kappa) has been added to the bottom of the table. In red, we highlight the similarities. TVUS, transvaginal ultrasound. Internal adenomyosis (TVUS) means close to the endometrium; external adenomyosis (TVUS) means lesions that affect the outer myometrium/subserosa.

**Table 3:** Comparison between histological and ultrasound aspects of external adenomyosis.

Pattern	*p* Value	Histological Location		Location TVUS		Total
				External	Internal	
Diffuse	*p* = 0.0001	External (deep) histology	No	0 (0.0%)	45 (90.0%)	45 (54.9%)
			Yes	32 (100.0%)	5 (10.0%)	37 (45.1%)
		Total		32 (100.0%)	50 (100.0%)	82 (100.0%)
Focal	*p* = 0.0001	External (deep) histology	No	11 (29.7%)	40 (97.6%)	51 (65.4%)
			Yes	26 (70.3%)	1 (2.4%)	27 (34.6%)
		Total		37 (100.0%)	41 (100.0%)	78 (100.0%)
Total	*p* = 0.0001	External (deep) histology	No	11 (15.9%)	85 (93.4%)	96 (60.0%)
			Yes	58 (84.1%)	6 (6.6%)	64 (40.0%)
		Total		69 (100.0%)	91 (100.0%)	160 (100.0%)
Weighted Kappa				0.781		
95% CI				0.684 to 0.879		

Inter-rater agreement (Kappa) has been added to the bottom of the table. In red, we highlight the similarities. TVUS, transvaginal ultrasound. Internal adenomyosis (TVUS) means close to the endometrium; external adenomyosis (TVUS) means lesions that affect the outer myometrium/subserosa.

**Table 4:** Symptoms of internal and external adenomyosis.

Symptoms	Internal			External			Total
	No	Yes	*p*-Value	No	Yes	*p*-Value	
Pelvic pain	76 (96.2%)	73 (90.1%)	*p* = 0.21	86 (89.6%)	63 (98.4%)	***p* = 0.030**	149 (93.1%)
Abnormal bleeding	70 (88.6%)	81 (100.0%)	***p* = 0.001**	91 (94.8%)	60 (93.8%)	*p* = 0.779	151 (94.4%)
Female infertility	8 (10.1%)	28 (34.6%)	***p* = 0.001**	35 (36.5%)	1 (1.6%)	***p* = 0.001**	36 (22.5%)
Leiomyoma	41 (51.9%)	49 (60.5%)	*p* = 0.33	54 (56.2%)	36 (56.2%)	*p* = 0.56	90 (56.2%)
Endometriosis	38 (48.1%)	1 (1.2%)	***p* = 0.001**	2 (2.1%)	37 (57.8%)	***p* = 0.001**	39 (24.4%)
Total	79 (100.0%)	81 (100.0%)		96 (100.0%)	64 (100.0%)		160 (100.0%)

We used red color for the statistically significant *p*-value.

**Table 5:** Association between symptoms and alteration of the junctional zone.

Symptoms	*p*-Value	Junctional Zone		
***p* = 0.047**		**JZ-Altered**		**Total**
		**No**	**Yes**	
Pelvic pain	No	0 (0.0%)	11 (9.2%)	11 (6.9%)
	Yes	40 (100%)	109 (90.8%)	149 (93.1%)
Total		40 (100%)	120 (100%)	160 (100%)
***p* = 0.0001**		JZ-altered		Total
		No	Yes	
Abnormal bleeding	No	7 (17.5%)	2 (1.7%)	9 (5.6%)
	Yes	33 (82.5%)	118 (98.3%)	151 (94.4%)
Total		40 (100%)	120 (100%)	160 (100%)
Crosstab				
***p* = 0.0001**		JZ-altered		Total
		No	Yes	
Female infertility	No	49 (98%)	75 (68.2%)	124 (77.5%)
	Yes	1 (2%)	35 (31.8%)	36 (22.5%)
Total		50 (100%)	110 (100%)	160 (100%)

JZ, junctional zone. A *p*-value < 0.05 is statistically significant, and we used the red color.

## Data Availability

The original contributions presented in this study are included in the article. Further inquiries can be directed to the corresponding author.

## Abbreviations

The following abbreviations are used in this manuscript:

TVUS	Transvaginal ultrasound
IP	Pulsatility index
MUSA	Morphological Uterus Sonographic Assessment
MRI	Magnetic resonance imaging
JZ	Junctional zone
AUB	Abnormal uterine bleeding
BMI	Body index mass
SIR	Society of Interventional Radiology
UAE	Uterine artery embolization
HIFU	Focused ultrasound with high intensity
